# The Complete Mitochondrial Genome of the Chicken Body Louse, *Menacanthus cornutus*, and Evolutionary Patterns of Extensive Gene Rearrangements in the Mitochondrial Genomes of Amblycera (Psocodea: Phthiraptera)

**DOI:** 10.3390/genes13030522

**Published:** 2022-03-16

**Authors:** Siyu Gong, Ye Xu, Shiwen Xu, Yanxin Liang, Li Tian, Wanzhi Cai, Hu Li, Fan Song

**Affiliations:** Department of Entomology and MOA Key Lab of Pest Monitoring and Green Management, College of Plant Protection, China Agricultural University, Beijing 100193, China; gsy1207@cau.edu.cn (S.G.); xuyezdk@163.com (Y.X.); xusw2019@126.com (S.X.); yusisama@163.com (Y.L.); ltian@cau.edu.cn (L.T.); caiwz@cau.edu.cn (W.C.); tigerleecau@hotmail.com (H.L.)

**Keywords:** *Menacanthus cornutus*, mitochondrial genome, gene rearrangement, evolution

## Abstract

Animal mitochondrial (mt) genomes are typically double-strand circular DNA molecules, but diverse structural variations have been widely found in multiple groups. In parasitic lice (Phthiraptera), the structure of mt genomes varies remarkably across all five suborders. In this study, we reported the complete mt genome of a chicken body louse, *Menacanthus cornutus*, which has a typical single circular mt chromosome and drastic mt gene rearrangements. This mt genome is 15,693 bp in length, consisting of 13 protein-coding genes, 23 tRNA genes, 2 rRNA genes, and a control region. A comparison with a typical insect mt genome suggested that two highly similar *trnM* are present in the mt genome of *M. cornutus*. Moreover, almost every single gene was rearranged, and over half of mt genes were inverted. Phylogenetic analyses inferred from the mt genome sequences supported the monophyly and position of Amblycera. Mapped over the phylogenetic relationships of Amblycera, we identified two inversion events for the conserved gene blocks in Boopidae and Menoponidae. The inverted *ND4L-ND4* was likely a synapomorphic rearrangement in Menoponidae. Our study demonstrated the importance of sequencing mt genomes for additional taxa to uncover the mechanism underlying the structural evolution of the mt genome in parasitic lice.

## 1. Introduction

The mitochondrion is an organelle with independent genetic material that is closely related to cellular energy metabolism and acts as a power bank in the life activities of eukaryotes [1,2,3]. Characterized by its stable gene composition, conserved gene order, maternal inheritance, and fast evolutionary rate [2,4], the mitochondrial (mt) genome has been extensively applied as a molecular marker for species delimitation, molecular phylogeny, population genetics and so forth [5,6,7,8]. Typically, an animal mt genome is a 14–20 kb double-strand circular DNA molecule containing 13 protein-coding genes (PCGs), 22 transfer RNA genes (tRNAs), two ribosomal RNA genes (rRNAs), and a control region (CR) regulating the replication and transcription of the mt genome [2,4,9]. The majority of insect mt genomes present a relatively conservative gene order, such as that in *Drosophila yakuba*, which is considered as the putative ancestral pattern of the insect gene arrangement [10] Yet, with a rapid increase in insect mt genome sequencing in recent years, structural variations with novel gene arrangement patterns have been discovered, especially in the order Phthiraptera [11,12,13,14,15], whose gene rearrangement is the most intense, with almost every single gene rearranged.

Phthiraptera, also known as parasitic lice, are mostly harmful parasitic insects. There are approximately 5000 species in five suborders, of which about 4000 and 1000 species parasitize birds and mammals, respectively [16,17]. In addition to the high degree of gene rearrangements, the typical single chromosome of the mt genome was found to be replaced by several circular minichromosomes in some parasitic lice, a phenomenon commonly known as mt genome fragmentation [18,19,20,21,22,23]. Mt genome fragmentation was first discovered in the human body louse, *Pediculus humanus*, whose typical single chromosome was fragmented into 20 minichromosomes [18,19]. The fragmented mt genomes were indicated to be an ancestral trait in a clade of Eutherian mammal lice (Trichodectera, Rhynchophthirina, and Anoplura) [20]. However, more recent studies suggest that mt genome fragmentation is more widespread among lice, as full or partial mt genomes with multiple chromosomes have been reported from Ischnocera [21] and Amblycera [22]. Moreover, there is a high degree of intra-genus variation in the patterns of gene rearrangements and genome fragmentation in lice [20,21], making this order an ideal group for exploring the evolutionary mechanism underpinning the structural evolution of the mt genome.

To date, a total of 37 complete or nearly complete mt genomes of parasitic lice have been published in the GenBank database. As the suborder with the most diverse host, which is parasitic on both birds and mammals, Amblycera plays a non-negligible role in the evolution of parasitic lice and may contain valuable evidence for the mt genome structural evolution. However, for Amblycera, which contains six families, only three complete mt genomes from two families (Boopidae and Menoponidae) were reported [11,20,22]. In this study, we applied high-throughput sequencing to obtain the mt genome of the chicken body louse *Menacanthus cornutus*, a pathogenic hematophagous species in Amblycera that may cause anaemia, heavy multi-focal lesion, or even the death of infested birds [24,25]. We analyzed the patterns of gene rearrangement and genome fragmentation in this species, and we used the mt gene data to recover the phylogenetic relationships of Phthiraptera. Based on the phylogenetic tree, we assessed the evolution of gene arrangements in Amblycera. Our study enriched the mt genome data of Amblycera and provided more information to the diversity and evolution of louse mt genomes.

## 2. Materials and Methods

### 2.1. Sample Collection, Genomic DNA Extraction, and Sequence Amplification

Louse specimens were collected from chickens, *Gallus gallus* in Chongqing Jinyun Mountain, which acted as the host of *Menacanthus cornutus*. The specimens were preserved in 100% ethanol at −20 °C after removal from their host until DNA extraction. Total genomic DNA was extracted from a whole individual louse using the DNeasy Blood and Tissue Kit (QIAGEN, Hilden, Germany) following the manufacturer’s instructions. The genomic DNA was stored below –20 °C at the Entomological Museum of China Agricultural University (Beijing, China).

The fragments of two mt genes (*COI*, ~610 bp and *srRNA*, ~530 bp) were amplified by polymerase chain reaction (PCR). The PCR products were sequenced by Sanger sequencing at Tsingke Biotechnology (Beijing, China).

### 2.2. Mitochondrial Genome Sequencing, Assembly, and Annotation

An Illumina TruSeq library was prepared with an average insert size of 350 bp and was sequenced on the Illumina NovaSeq 6000 platform at Berry Genomics (Beijing, China). A total of 8 Gb of clean data (150 bp paired-end reads) was obtained for the species. The raw reads were trimmed of adapters using Trimmomatic [26] and low-quality and short reads were removed using Prinseq [27]. High-quality reads were used in *de novo* assembly using IDBA-UD [28] with the parameters: similarity threshold 98%, minimum *k* value 45, and maximum *k* value 145. To identify the mt genome sequence, the assembled contigs were searched with the *COI* and *srRNA* partial sequences using BLAST [29] with at least 98% similarity. The contigs were then used as reference sequences to fish sequencing reads in Geneious Prime v2020.0.5 using the “map to reference” option. The assembly parameters were set as no gaps, minimum overlap 100 bp, minimum overlap identity 98%, maximum mismatches per read 2%, and maximum ambiguity 2 until the two ends of the contig overlapped and ensured the graph of coverage was smooth. Finally, we obtained a complete circular mt genome with an average coverage depth of 2901×.

tRNA genes were annotated using the MITOS web server [30] and the tRNAscan-SE search server [31] with the invertebrate mt code. The locations of PCGs and rRNA genes were identified by alignment with homologous genes of other parasitic lice available in the GenBank. The nucleotide composition of the mt genome was analyzed with MEGA 11 [32]. To measure the nucleotide compositional differences of the whole mt genome, we also used the following formulas: (1) AT skew = (A − T)/(A + T) and (2) GC skew = (G − C)/(G + C) to calculate the AT- and GC-skews [33].

### 2.3. Phylogenetic Analyses

A total of 32 mt genomes of parasitic lice were used for the phylogenetic analyses, including the newly sequenced mt genome of *Menacanthus cornutus*. Seven mt genomes (one Hemiptera species and six Psocoptera species) were used as outgroups (Table 1).

Eleven PCGs and two rRNA genes were used for phylogenetic analyses. The *ND2* and *ND6* genes were not found in the elephant louse (*Haematomyzus elephantis*) and opossum louse (*Cummingsia maculata*), and, thus, these two PCGs were excluded from the phylogenetic analyses. Each PCG was aligned individually based on codons for amino acids using the MAFFT algorithm [34], implemented in the TranslatorX online platform with the L-INS-i strategy and default setting [35]. Two rRNA genes were individually aligned using the MAFFT v7.0 online server with the G-INS-i strategy [36]. After removing poorly aligned sites using GBlocks v0.91b [37], the alignment of the individual gene was concatenated into two datasets: (i) the PCGRNA matrix, including eleven PCGs and two rRNA genes (7815 bp in total) and (ii) the PCG12RNA matrix, including the first and second codon positions of the eleven PCGs and two rRNA genes (5689 bp in total).

Many previous studies have revealed that the site-heterogeneous mixture model (CAT+GTR) implemented in PhyloBayes can avoid the false grouping of unrelated taxa with a similar base composition and an accelerated evolutionary rate in the reconstruction of the high-level phylogeny of many insect orders based on mt genome sequences [6,7,38,39]. Therefore, both datasets were analyzed under the CAT+GTR model using PhyloBayes MPI v1.8c [40]. Two independent Markov chain Monte Carlo chains were run after the removal of constant sites from the alignment and were stopped after the two runs had satisfactorily converged (maxdiff < 0.1). A consensus tree was computed from the remaining trees that were combined from two runs after the initial 25% trees of each run were discarded as burn-in.

## 3. Results and Discussion

### 3.1. The General Features of the Menacanthus cornutus mt Genome

**Figure 1:**
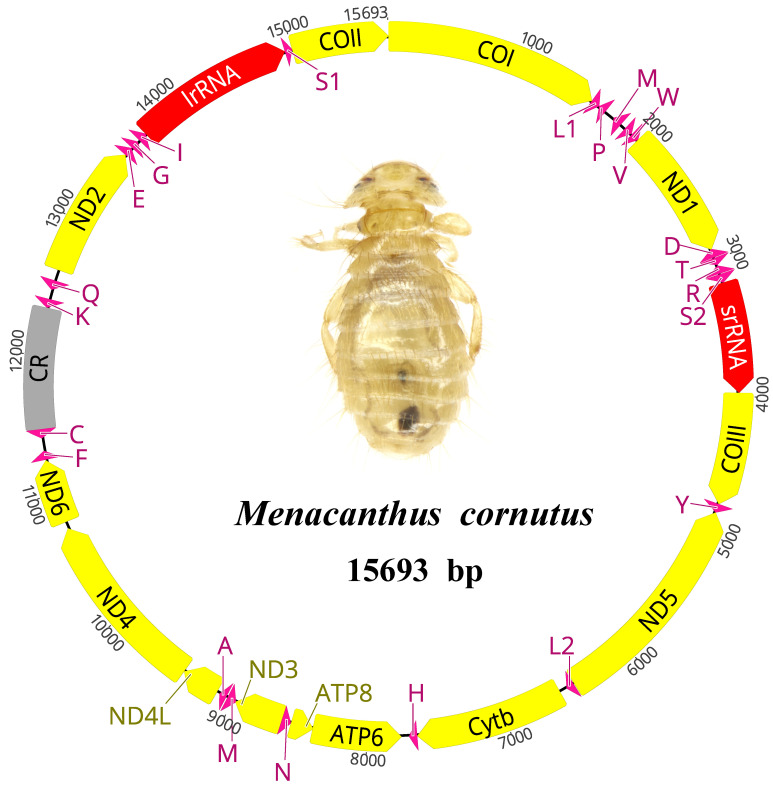
The mitochondrial genome of *Menacanthus cornutus*. The arrows indicate the direction of gene transcription. The yellow, pink, red, and gray colors indicate the regions of PCGs, tRNA genes, rRNA genes, and control regions, respectively. Abbreviations: *ATP6* and *ATP8*, adenosine triphosphate (ATP) synthase subunits 6 and 8; *COI–COIII*, cytochrome C oxidase subunits I-III; *CytB*, cytochrome b; *ND1-ND6* and *ND4L*, nicotinamide adenine dinucleotide hydrogen (NADH) dehydrogenase subunits 1-6 and 4L; *lrRNA* and *srRNA*, large and small rRNA subunits; The tRNA genes are indicated with their one-letter corresponding amino acids; CR, control region.

The complete mt genome of *Menacanthus cornutus* is a single circular chromosome that is 15,693 bp in size, including 38 coding genes (13 PCGs, 23 tRNA genes, and 2 rRNA genes) and a CR (Figure 1, Table S1). In total, 12 genes were transcribed from the minority strand (N-strand) while the other 26 genes were encoded on the majority strand (J-strand). Six gene overlaps were observed, ranging from 1 to 7 bp in length, and the longest occurred between *ATP6* and *ATP8,* which was common among the mt genomes of insects [4]. The CR was located between *trnC* and *trnK,* with a size of 871 bp. Apart from the CR, we also identified 23 non-coding regions (NCRs) comprising a total of 582 bp, with the longest being 88 bp between *trnC* and *trnF*.

All the PCGs were initiated by ATN (eight by ATT, two by ATA, one by ATA, and one by ATC), except *ND1* used TTG as a start codon. A complete TAA or TAG was used by all the PCGs as the termination codon (Table S1). The 23 detected tRNA genes ranged from 54–70 bp, and all the secondary structures could be folded into the typical clover-leaf structure. Different from the typical 22 tRNAs in insect mt genomes, 2 *trnM* were found in the *Menacanthus cornutus* mt genome. The sequences of the two *trnM* were highly identical, with differences occurring on only six nucleotide positions in the DHU arm and the TΨC arm (Figure S1). Two copies of *trnM* were also detected in *Cummingsia maculata* (Amblycera: Trimenoponidae), whereas the sequence similarity between the two *trnM* was lower than *Menacanthus cornutus* (16 nucleotide positions were different). The large rRNA subunit (*lrRNA*) and small rRNA subunit (*srRNA*) were encoded on the J strand; however, these two rRNA genes were transcribed from the N strand in the ancestral insect mt genome [4,41]. The two rRNA genes were in the locations *trnI* to *trnS1* and *trnS2* to *COIII*, with lengths of 1163 bp and 800 bp respectively.

The nucleotide composition of the *Menacanthus cornutus* mt genome is significantly biased toward adenine (A) and thymine (T), with an A+T content of 74.1% (Table S2). In contrast to most published insect mt genomes, the nucleotide composition shows a negative AT-skew (−0.055) and a positive GC-skew (0.023) [42]. The AT and GC skews of several amblyceran lice are all negative, which may result from the gene rearrangement events, especially the inversion of genes [22]. Within the whole mt genome, CR shows the most biased A+T content (76.9%), while the A+T content is 73.8% in the PCGs, 73.1% in the tRNAs, and 73.7% in the rRNAs, respectively.

### 3.2. Phylogenetic Analyses of the Suborder Amblycera and Other Parasitic Lice

**Figure 2:**
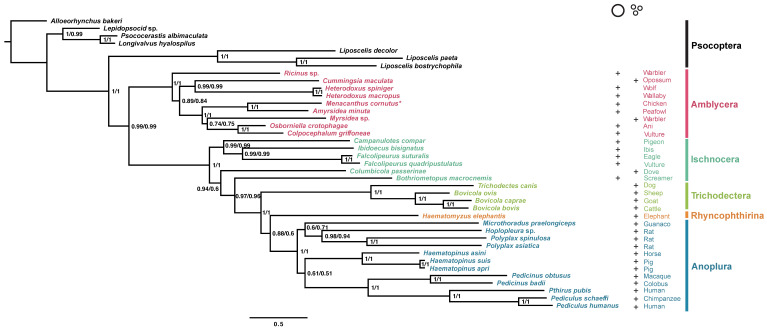
Phylogenetic trees of Phthiraptera inferred from the PCGRNA and PCG12RNA datasets using PhyloBayes under the site-heterogeneous mixture CAT + GTR model. Supports at nodes (from left to right) are Bayesian posterior probabilities (BPPs) for PCGRNA and PCG12RNA. The mitochondrial genome sequence of *M. cornutus* is highlighted with an asterisk (*). The genome architecture (typical or fragmented) is indicated by a plus sign (+). The bird and mammal hosts are indicated after the names of the parasitic lice.

The suborder-level phylogeny of Pththiraptera was reconstructed using Bayesian inference based on the two concatenated datasets (PCGRNA and PCG12RNA) (Figure 2). The two phylogenetic trees showed identical topology. The monophyly of the Phthiraptera was well supported (BPPs = 1), and the sister relationship between Phthiraptera and the family Liposcelididae was highly supported (BPPs = 1). Amblycera was monophyletic with strong support (BPPs = 1) and was relatively distantly related to the other monophyletic clade that included all four other suborders. The topology of the phylogenetic tree also supported the paraphyly of Ischnocera, as *Columbicola passerinae* and *Bothriometopus macrocnemis* were not nested inside other ischnoceran species. However, this bird louse clade was shown to be monophyletic by studies using nuclear genes [43,44]. Trichodectera, which was previously regarded as the family Trichodectidae in Ischnocera, was clustered with Rhynchophthirina and Anoplura, which are parasitic on eutherian mammals, with strong support in our study (BPPs = 1). This mammal-infesting clade, comprising Trichodectera, Rhynchophthirina and Anoplura, was also supported to exist by recent studies using transcriptome data [43], shared derived features of mt genome fragmentation [20], and orthologous nuclear genes [44]. Within the suborder Amblycera, *Menacanthus cornutus* is indicated to be a sister lineage with *Amyrsidea minuta* (BPPs = 1), and a monophyletic Menoponidae was also well supported (BPPs = 1). Ricinidae was the most distantly related of the Amblycera species examined (BPPs = 1), and the three mammal lice (*Cummingsia maculata*, *Heterodoxus macropus*, and *Heterodoxus spiniger*) were clustered together as the sister group to the family Menoponidae (BPPs = 0.99).

The published mt genomes of Trichodectera, Rhynchophthirina, and Anoplura lice, which are parasitic on eutherian mammals, all have extensive mt genome fragmentation (9–20 minichromosomes) [20,23]. The pigeon and dove louse *Columbicola passerinae*, from the family Philopteridae, was fragmentated into 15–17 minichromosomes [21]. In a recent study, fragmented mt genomes of Amblycera (*Cummingsia maculate* and *Myrsidea* sp.) were assembled from the sequencing data in the NCBI SRA database [22]. According to the phylogenetic trees in our study, the fragmented mt genomes evolved from single chromosome mt genomes more than once in Phthiraptera. Even in the suborder Amblycera, mt genome fragmentation evolved independently at least two times, as the *Cummingsia* maculate and *Myrsidea* sp. which have fragmented mt genomes were not nested in one clade.

### 3.3. Evolution of Gene Rearrangement in the Mitochondrial Genome of Amblycera

**Figure 3:**
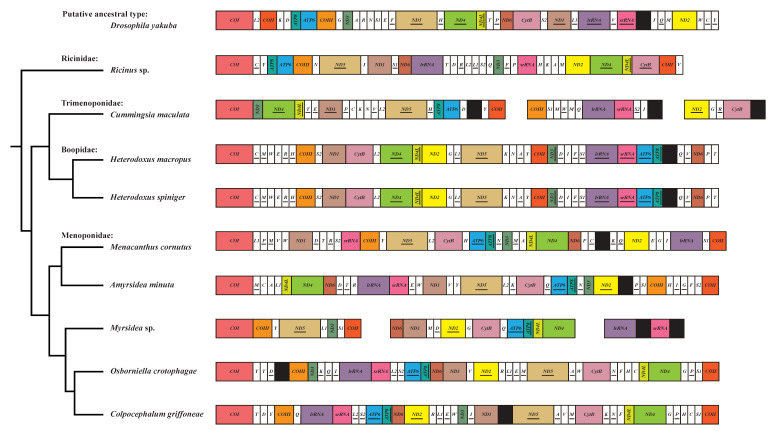
Gene arrangements of the Amblycera mitochondrial genomes. The mt genomes have been linearized for ease of comparison and arbitrarily begin with *COI* when possible. Different PCGs and rRNA genes are shown in different colors, with tRNA genes in white and control or non-coding intergenic regions in black. The gene names are the standard abbreviations used in this study. Underlined labels indicate the gene was transcribed from the minority strand.

Previous studies show that the mt genome of the order Phthiraptera is characterized by drastic mt gene rearrangement [11,12,13,14,15]. As one of the suborders of Phthiraptera, Amblycera also have variable and highly rearranged mt genomes [22]. For the newly sequenced mt genome of *Menacanthus cornutus*, nearly all genes were rearranged (Figure 3). No conserved gene clusters could be found in the tRNAs, and, in total, 12 tRNAs were inverted. Even for the PCGs and rRNAs, which rarely rearranged in insect mt genomes, only two conserved gene blocks could be detected (*ATP8-ATP6* and *ND4-ND4L*). However, the coding direction of the two gene blocks was inverted in the mt genome of *Menacanthus cornutus* compared to the putative ancestral type of *D. yakuba*.

To better understand the evolution of extensive gene rearrangements in the mt genomes of Amblycera, gene orders of published amblyceran mt genomes were summarized and mapped on an estimated phylogenetic tree (Table 2, Figure 3). The patterns of gene rearrangement are highly variable across Amblycera, and the gene blocks *ATP8-ATP6* and *ND4-ND4L* are the only region shared by all nine species. However, even in this region, a slight variation in the coding direction can be found between some species. In contrast to the other three families, an inverted *ND4L-ND4* is shared by all five Menoponidae species, and this gene rearrangement pattern may be synapomorphic across Menoponidae. The gene blocks *ATP8-ATP6* were not changed in Ricinidae and Trimenoponidae but were inverted in Boopidae and Menoponidae species. Such gene rearrangement patterns agree with the recovered phylogenetic relationships, as the outgroup, Ricinidae, and some of the ingroup, Trimenoponidae, had the same coding direction of *ATP8-ATP6* and *ND4-ND4L* as that found in *D. yakuba*, and two inversion events happened in Boopidae (*ATP8-ATP6* to *ATP6**-ATP8*) and Menoponidae (*ND4-ND4L* to *ND4L-ND4*) during the evolution of Amblycera. At the family level, we found that the gene blocks *trnS1-COII* and *lrRNA-srRNA* were present in most of Menoponidae species but disappeared in other families. In Boopidae, *Heterodoxus macropus,* and *Heterodoxus spiniger* have a completely identical gene order, indicating this gene arrangement may be conserved within the genus of Amblycera. The structure of the mt genome is more conversed in Menoponidae. *Colpocephalum griffoneae* and *Osborniella crotophagae* share identical gene arrangements in two major gene (PCGs and rRNAs) clusters *lrRNA-srRNA-ATP6-ATP8-ND6* and *ND5-Cytb-ND4L-ND4-COII-COI-COIII*.

Overall, our study added more data on the mt genomes of parasitic lice and enhanced our understanding of the structural diversity of the mt genome of lice. More mt genome sequencing and comparative studies are needed to uncover the mechanisms underlying the structural evolution of the mt genome in parasitic lice.

## Figures and Tables

**Table 1:** Taxa used in this study.

Order	Suborder	Family	Species	GenBank Accession Number
Outgroup				
Hemiptera	Heteroptera	Nabidae	*Alloeorhynchus bakeri*	NC_016432
Psocoptera	Trogiomorpha	Lepidopsocidae	*Lepidopsocid* sp.	NC_004816
	Psocomorpha	Psocidae	*Psococerastis albimaculata*	NC_021400
			*Longivalvus hyalospilus*	JQ910986
	Troctomorpha	Liposcelidae	*Liposcelis decolor*	NC_023839
			*Liposcelis bostrychophila*	JN645275-645276
			*Liposcelis paeta*	KF649225-649226
				
Ingroup				
Phthiraptera	Amblycera	Boopidae	*Heterodoxus macropus*	AF270939
			*Heterodoxus spiniger*	MW199168
		Menoponidae	*Menacanthus cornutus* ^a^	OM718871
			*Colpocephalum griffoneae*	NC_039530
			*Amyrsidea minuta*	MH001227
			*Osborniella crotophagae*	MW199175
			*Myrsidea* sp.	MW199172-199174
		Ricinidae	*Ricinus* sp.	MW199176
		Trimenoponidae	*Cummingsia maculata*	MW199177-199179
	Ischnocera	Trichodectidae	*Bovicola bovis*	MH001189-001200
			*Bovicola ovis*	MH001201-001212
			*Bovicola caprae*	MH001176-001188
			*Trichodectes canis*	MH001213-001224 and MH823541
		Philopteridae	*Ibidoecus bisignatus*	NC_015999
			*Bothriometopus macrocnemis*	EU183542
			*Campanulotes compar*	MH001225
			*Falcolipeurus quadripustulatus*	MH001226
			*Falcolipeurus suturalis*	MW696813
			*Columbicola passerinae*	MT094266-094282
	Rhyncophthirina	Haematomyzidae	*Haematomyzus elephantis*	KF933032–933041
	Anoplura	Polyplacidae	*Polyplax spinulosa*	KF647762–647772
			*Polyplax asiatica*	KF647751–647761
		Hoplopleuridae	*Hoplopleura* sp.	MT792483-792494
		Microthoraciidae	*Microthoradus praelongiceps*	KX090378–090389
		Haematopinidae	*Haematopinus asini*	KF939318, KF939322, KF939324, KF939326, and KJ434034–38
			*Haematopinus apri*	KC814611–814619
			*Haematopinus suis*	KC814602–814610
		Pthiridae	*Pthirus pubis*	EU219987–219995, HM241895–241898, JQ976018, and MT721740
		Pediculidae	*Pediculus schaeffi*	KC241882–241897
			*Pediculus humanus*	FJ499473–499490
			*Pedicinus obtusus*	MT792495–792506
			*Pedicinus badii*	MT721726–721739

^a^ Mitochondrial genome sequenced in the present study.

**Table 2:** The derived mitochondrial gene blocks shared by parasitic lice in Amblycera.

Family	Species	Fragmented mt Genome	*ND4-ND4L*	*ND4L-ND4*	*ATP8-ATP6*	*ATP6-ATP8*	*trnS1-COII*	*lrRNA-srRNA*
	*Drosophila yakuba* (Putative ancestral type)	−	**+**	**−**	+	−	−	−
Ricinidae	*Ricinus* sp.	−	**+**	**−**	+	−	−	−
Trimenoponidae	*Cummingsia maculata*	+	**+**	**−**	+	−	−	+
Boopidae	*Heterodoxus macropus*	−	**+**	**−**	−	+	−	−
	*Heterodoxus spiniger*	−	**+**	**−**	−	+	−	−
Menoponidae	*Menacanthus cornutus*	−	**−**	**+**	−	+	+	−
	*Amyrsidea minuta*	−	**−**	**+**	−	+	−	+
	*Myrsidea* sp.	+	**−**	**+**	−	+	+	+
	*Osborniella crotophagae*	−	**−**	**+**	−	+	+	+
	*Colpocephalum griffoneae*	−	**−**	**+**	−	+	+	+

Note: Plus (+) indicates presence; minus (−) indicates absence. Underlined labels indicate the gene was transcribed from the minority strand.

## Data Availability

The mitochondrial genome sequences of *Menacanthus cornutus* which used in this study have been deposited into GenBank (accession numbers: OM718871).

## Supplementary Materials

The following supporting information can be downloaded at: https://www.mdpi.com/article/10.3390/genes13030522/s1, Table S1: Structure of the *Menacanthus cornutus* mitochondrial genome, Table S2: Nucleotide composition of the *Menacanthus cornutus* mitochondrial genome, Figure S1: Secondary structures and sequence similarity of *trnM1* and *trnM2*. Inferred Watson-Crick bonds are illustrated by lines and GU bonds are illustrated by dots. The identical nucleotides between *trnM1* and *trnM2* are labeled with green circles and the variable sites are in pink.
