# Supplementary material for: The Complete Mitochondrial Genome of the Chicken Body Louse, Menacanthus cornutus, and Evolutionary Patterns of Extensive Gene Rearrangements in the Mitochondrial Genomes of Amblycera (Psocodea: Phthiraptera)
Source: Genes (Basel). 2022 Mar 16;13(3):522. doi: 10.3390/genes13030522 (PMC8950984; doi:10.3390/genes13030522)
Supplement: Supplementary file 1 [file genes-13-00522-s001.zip › genes-1633830-supplementary.pdf]

Supplementary materials

Table S1. Structure of *Menacanthus cornutus* mitochondrial genome.

Gene	Strand	Position	Size (bp)	Start Codon	Stop Codon	Anticodon	Intergenic Nucleotide
COI	+	1-1548	1548	ATT	TAG		-5
trnL1	+	1547-1610	64			TAG	-2
trnP	-	1612-1668	57			TGG	1
trnM1	-	1745-1814	70			CAT	76
trnV	+	1857-1914	58			TAC	42
trnW	+	1919-1979	61			TCA	4
ND1	+	1980-2921	942	TTG	TAA		0
trnD	-	2919-2982	64			GTC	-3
trnT	+	2986-3043	58			TGT	3
trnR	-	3047-3106	60			TCG	3
trnS2	+	3110-3172	63			TGA	3
srRNA	+	3173-3972	800				0
COIII	+	3973-4779	807	ATT	TAG		0
trnY	+	4778-4841	64			GTA	-2
ND5	-	4842-6455	1614	ATT	TAG		0
trnL2	+	6457-6518	62			TAA	1
CytB	+	6569-7642	1074	ATA	TAA		50
trnH	+	7646-7705	60			GTG	3
ATP6	-	7755-8396	642	ATG	TAA		49
ATP8	-	8390-8554	165	ATT	TAA		-7
trnN	-	8555-8622	68			GTT	0
ND3	+	8624-8977	354	ATT	TAA		1
trnM2	-	8975-9043	69			CAT	-3
trnA	+	9053-9106	54			TGC	9
ND4L	+	9128-9403	276	ATT	TAA		21
ND4	+	9405-10730	1326	ATA	TAA		1
ND6	+	10783-11244	462	ATC	TAA		52
trnF	+	11249-11314	66			GAA	4
trnC	-	11403-11466	64			GCA	88
CR		11467-12337	871				0
trnK	-	12338-12400	63			TTT	0
trnQ	-	12472-12538	67			TTG	71
ND2	+	12623-13576	954	ATT	TAA		84
trnE	+	13578-13641	64			TTC	1
trnG	+	13644-13705	62			TCC	2
trnI	+	13719-13783	65			GAT	13
lrRNA	+	13784-14946	1163				0
trnS1	+	14947-15002	56			TCT	0
COII	+	15003-5	696	ATT	TAA		0

Table S2. Nucleotide composition of *Menacanthus cornutus* mitochondrial genome.

	A%	C%	G%	T(U)%	AT%	AT-Skew	GC-Skew	size (bp)
Whole genome	35.0	12.6	13.2	39.1	74.1	-0.055	0.023	15693
Control region	36.1	11.6	11.6	40.8	76.9	-0.061	0.000	871
PCGs	31.4	12.7	13.5	42.4	73.8	-0.149	0.031	10860
PCGs (J)	31.4	13.4	13.6	42.2	73.6	-0.147	0.007	8439
PCGs (N)	32.5	11.2	13.1	43.2	75.7	-0.141	0.078	2421
tRNAs	39.0	12.2	14.7	34.1	73.1	0.067	0.093	1439
tRNAs (J)	37.9	12.7	15.8	33.6	71.5	0.060	0.109	857
tRNAs (N)	40.5	11.3	13.2	34.9	75.4	0.074	0.078	582
rRNAs	39.3	11.2	15.1	34.4	73.7	0.066	0.148	1963

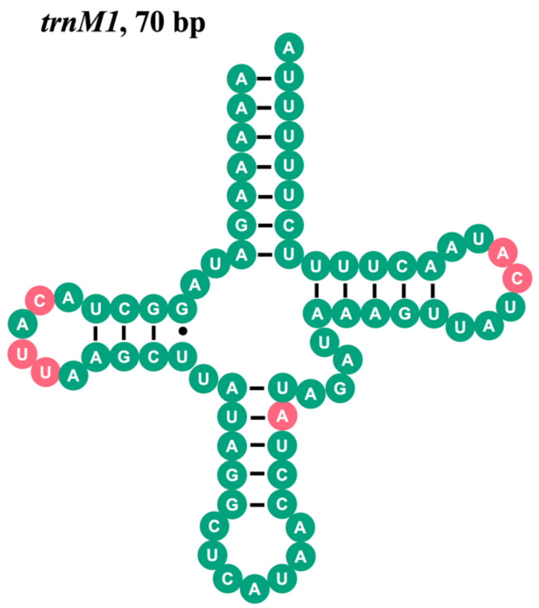

Figure S1. Secondary structures and sequence similarity of *trnM1* and *trnM2*. Inferred Watson-Crick bonds are illustrated by lines and GU bonds are illustrated by dots. The identical nucleotides between *trnM1* and *trnM2* are labeled with green circles and the variable sites are in pink.
